# Post-COVID-19 syndrome: Insights into a novel post-infectious systemic disorder

**DOI:** 10.25122/jml-2022-0329

**Published:** 2023-02

**Authors:** Marilena Stoian, Bianca Procopiescu, Silviu Șeitan, Gabriel Scarlat

**Affiliations:** 1 Department of Internal Medicine, Dr. Ion Cantacuzino Clinical Hospital, Bucharest, Romania; 2 Carol Davila University of Medicine and Pharmacy, Bucharest, Romania

**Keywords:** COVID-19, post-COVID-19 syndrome, systemic inflammatory syndrome, immune system

## Abstract

Coronavirus disease 2019 (COVID-19) is currently considered a complex systemic infectious and inflammatory disease, determined by the infection with severe acute respiratory syndrome coronavirus-2 (SARS-CoV-2), and the cause of one of the most important epidemiological phenomena in the last century – the COVID-19 pandemic. This infectious-inflammatory disease may generate a wide range of clinical manifestations and biological modifications, explained by the ubiquitous nature of the SARS-CoV-2 receptors, represented by the angiotensin-converting enzyme-2 (ACE-2), and by the host’s violent immune and proinflammatory reaction to the viral infection. These manifestations include immunological disturbances, which, according to certain clinical findings, may persist post-infection, in the form of a presumed systemic inflammatory entity, defined by several clinical concepts with a common pathological significance: post-COVID-19 multisystem (or systemic) inflammatory syndrome, post-COVID syndrome or long-COVID. Although the pathophysiological mechanisms of the post-COVID-19 syndrome are elusive at the present moment, there are currently several studies that describe a systemic inflammatory or autoimmune phenomenon following the remission of the COVID-19 infection in some patients, which suggests the existence of molecular and cellular immune abnormalities, most probably due to the host’s initial violent immune response to the viral infection, in the form of three overlapping entities: secondary hemophagocytic lymph histiocytosis (HLH), macrophage activation syndrome (MAS) and cytokine release syndrome (CRS). Thus, this is reminiscent of different classic autoimmune diseases, in which various infections are risk factors in developing the autoimmune process.

## INTRODUCTION

### The association between the main immunopathological aspects of COVID-19 and post-COVID-19 syndrome

Based on our personal clinical experience and various clinical findings [1-4], post-COVID syndrome (PCS), also known as post-COVID-19 multisystem (systemic) inflammatory syndrome or long-COVID, may be defined as a polymorphic clinical and pathophysiological entity characterized by persistent or recurrent clinical manifestations and biological modifications. Most of these symptoms are indicative of an inflammatory disorder developed by some individuals after the remission of SARS-CoV-2 infection, in the absence of other infections and other associated pathological conditions. Hence, PCS may be considered a chronic immunoinflammatory complication of COVID-19, governed by a latent hyperinflammatory state. Even though this particular syndrome does not currently have a well-established and specific definition, thus requiring refinement, its description is solely based on its clinical and biological presentation.

Although the pathophysiological mechanisms by which PCS develops post-infection are currently not well understood, it may be assumed that this entity could develop as a consequence of an immunological dysregulation or as a resultant hyperinflammatory state previously induced by the infection with SARS-CoV-2 associated with the host’s complex dysregulated immunoinflammatory reaction. This assumption is based on several clinical findings, including our own [1,5,6], in which a hyperinflammatory or potential autoimmune phenomenon may be a fundamental pathophysiological aspect. Such a systemic disturbance could be at least partially explained by the presence of various immunoinflammatory phenomena that certain patients manifest in the context of moderate and, especially, severe forms of COVID-19, in which there is apparent immune hyperactivity associated with hyperactivation of macrophages and T-lymphocytes and uncontrolled systemic secretion of various chemokines and proinflammatory cytokines, such as interleukin-1β (IL-1β), interleukin-6 (IL-6), interferon-γ (IFN-γ), tumor-necrosis factor-α (TNF-α), granulocyte-monocyte colony-stimulating factor (GM-CSF) and macrophage inflammatory protein-1α (MIP-1α), this hyperinflammatory milieu being known as cytokine release syndrome (CRS) or cytokine storm [7]. CRS is most frequently associated with hyperferritinemic syndromes, a pathophysiological concept encompassing several entities: MAS, which is considered a subset of HLH, adult-onset Still’s disease (AOSD), catastrophic antiphospholipid syndrome (CAPS) and septic shock. All of these entities are characterized by dramatic elevation of serum ferritin (hyperferritinemia) and a systemic hyperinflammatory state induced by the cytokine storm, which may determine multiple organ dysfunction syndrome (MODS)[8]. Over the last two years, extensive research and clinical and laboratory findings have proved that some severe forms of COVID-19 share several features with the pathological conditions, which currently place COVID-19 in the spectrum of hyperferritinemic syndromes [9].

The association between secondary hemophagocytic lymph histiocytosis (sHLH) and COVID-19 is noteworthy, as it has been discussed and researched since 2020. HLH is a rare and potentially fatal hyperinflammatory syndrome that shares similarities with MAS and CRS and is characterized by hyperactivation and overproduction of macrophages (histiocytes) and lymphocytes in various tissues, including lymphoid organs (lymph nodes, spleen, bone marrow), along with excessive release of proinflammatory molecules [10-13]. Although its pathogenic mechanisms are not entirely understood, studies have shown the existence of defective granule-mediated cytotoxicity and uncontrolled T-lymphocyte activation, which induces a systemic hyperinflammatory state associated with multiple tissue involvement and progression toward MODS [14]. The typical clinical presentation of HLH consists of recurrent high fever (>38℃), hepatosplenomegaly, and progressive multiple organ damage, including acute liver failure, whereas laboratory findings include marked elevation of C reactive protein (indicative of a non-specific inflammatory syndrome), hyperferritinemia (suggesting a hyperferritinemic syndrome), cytopenia’s (bicytopenia or pancytopenia), hypertriglyceridemia, increased serum level of liver enzymes, hemophagocytosis detected on bone marrow biopsy [11] and, according to a case report from 2014, marked histiocytosis engulfing lymphocytes and red blood cells on lymph node biopsy [15]. Whereas primary HLH (pHLH) is hereditary (familial) and mainly affects children [16], sHLH is an acquired, frequently unrecognized hyperinflammatory syndrome affecting patients of all ages, including adults. It may be generated by various viral infections (mainly Epstein-Barr virus), autoimmune and autoinflammatory diseases (systemic lupus erythematosus, juvenile idiopathic arthritis, AOSD), and several malignancies (mainly hematological malignancies, such as leukemias, Hodgkin lymphoma, and non-Hodgkin lymphomas) [17-20].

Although the pathophysiological relationship between severe COVID-19 and sHLH is not well-defined, a series of studies suggest that some patients with severe COVID-19 could develop sHLH, these patients exhibiting a hyperinflammatory state, like that of sHLH of other causes, such as elevated C reactive protein, hyperferritinemia, altered liver function, and coagulopathy, expressed by increased serum levels of D-dimers [21-24]. Moreover, a study performed by Claudia Núñez-Torrón *et al*., published in the Journal of Clinical Pathology in 2021, to ascertain the existence of sHLH in several patients with severe COVID-19 showed that eleven out of sixteen patients (68,7%) expressed moderate histiocytosis associated with hemophagocytosis in the bone marrow, which is indicative of COVID-19-associated sHLH [25].

The HLH-2004 study, involving 369 patients, demonstrated a range of clinical and biological characteristics, thereby serving as diagnostic criteria for HLH [26]:

- Fever ≥38.5℃ (in 95% of cases);
- Splenomegaly (in 89% of cases);
- Peripheral blood cytopenia, with the following associated modified parameters: hemoglobin levels <9 g/dl, platelet count <100,000/microL, absolute neutrophil count <1000/microL (in 92% of cases);
- Hypertriglyceridemia (with fasting triglycerides >265 mg/dL) and/or hypofibrinogenemia (with a fibrinogen level <150 mg/dL) (in 90% of cases);
- Hemophagocytosis in bone marrow, spleen, lymph nodes, or liver (in 82% of cases);
- Low or absent NK-cell activity (in 71% of cases);
- Hyperferritinemia (with a serum ferritin level >500 ng/mL) (in 94% of cases);
- Elevated soluble CD25 (soluble IL-2-receptor-α, sIL-2R).

The diagnosis of HLH is established if at least 5 of these 8 criteria are fulfilled. HLH initially manifests as a febrile syndrome associated with multiple organ involvement and modifications of biological parameters, which proves that the clinical presentation of HLH may mimic various infections or fever of unknown origin [27].

There have also been two reports of an atypical sHLH in post-COVID-19 patients – one of a 40-year-old female and one of a 2-year-old male –which is a noteworthy aspect regarding the nature of post-COVID-19 inflammatory syndrome in some individuals exhibiting a hyperinflammatory state. Although rare, immunological dysregulation following COVID-19 remission in some patients is the main reason for sHLH in such patients [28].

Furthermore, some studies explain the existence of an autoimmune state in severe COVID-19 patients, although this disease is not considered an autoimmune disorder per se. Some COVID-19 patients develop several autoantibodies during the disease [29,30]. These autoantibodies are directed against 12 host antigens, some of these immune molecular elements being antinuclear antigen antibodies (ANA), anti-SSA/Ro antibodies, anti-Scl-70 antibodies, and anti-U1-RNP antibodies [29]. According to studies, none of the patients in which the autoimmune phenomenon was detected had a medical history of systemic autoimmunity, even though approximately 70% of these patients expressed autoantibodies, which were related to various systemic autoimmune rheumatic diseases [30]. Immune thrombocytopenia was also detected in some COVID-19 patients, characterized by a biologically evident decrease in platelet count, explained by the existence of autoantibodies directed to the circulating platelets [31,32]. In addition, there are reports regarding viral-induced autoimmunity associated with COVID-19, the mechanism of molecular mimicry being well-described, as some proteins found on the surface of SARS-CoV-2 are biochemically identical to some proteins described in some host cells [33,34]. This suggests that some patients with COVID-19 may exhibit cross-reactive immunological reactions similar to those described in other pathological conditions, such as acute post-streptococcal glomerulonephritis or rheumatic endocarditis. Hence, the possible autoimmune phenomena occurring during the evolution of COVID-19 or post-COVID-19 are defined because of immune dysregulation during SARS-CoV-2 infection [35].

## PATHOPHYSIOLOGY: RECENT STUDIES

The phenomenon of the post-COVID-19 syndrome was first documented during 2020 and early 2021, as clinical evidence indicated that certain individuals, including children and adults previously infected with SARS-CoV-2, persisted in presenting symptoms and laboratory alterations well after the remission of the viral infection. Physicians initially defined this pathological condition as “long-term COVID-19”, which describes a complex of signs, symptoms, and modified biological parameters in individuals who had been previously diagnosed with COVID-19 but did not completely recover from the disease post-remission, exhibiting clinical manifestations for more than three weeks or several months [36]. However, this particular and somewhat obscure and polymorphic condition is currently described in the medical literature under various names but having the same clinicopathological significance: long-COVID, post-COVID-19 inflammatory syndrome, post-COVID syndrome (abbreviated as PCS), post-COVID-19 conditions.

**Figure 1:**
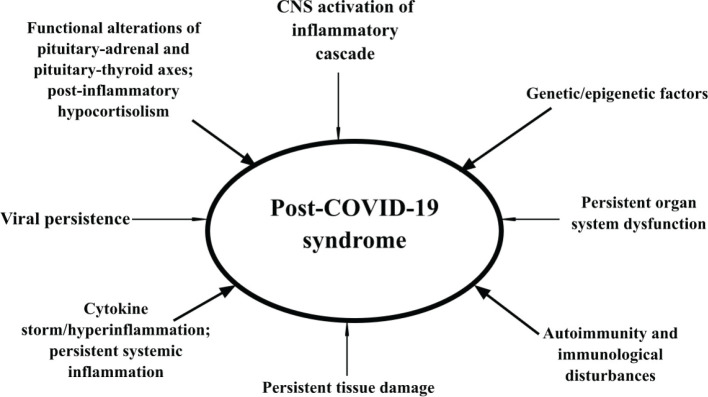
Possible pathophysiological mechanisms of the post-COVID-19 syndrome (PCS).

As shown in Figure 1, there are several possible pathophysiological mechanisms through which PCS may develop, although the immunopathological mechanisms and a potential functional alteration of the hypothalamic-pituitary-adrenal (HPA) axis are currently the most frequently discussed. It should also be noted that some studies, such as that published by Bektas A. *et al*. in 2020, describe that the possible pathophysiology of PCS is related mainly to hyperinflammatory states, oxidative stress, cytokine storm, and even DNA damage [37].

### Immunopathology of PCS

To properly understand the potential immunopathological nature of PCS, it is of great importance to reveal some autoimmune aspects described by several studies in some COVID-19 patients, mainly in those with severe forms of the disease.

Studies over the years have shown that some viruses, operating as environmental factors, may contribute to the development of some autoimmune phenomena through the increased production of autoantibodies by the host’s immune system. Such examples of viruses are Epstein-Barr virus (EBV), cytomegalovirus (CMV), and human immunodeficiency virus (HIV) [38]. SARS-CoV-2 may induce similar autoimmune manifestations, given that, according to recent studies, COVID-19 patients may develop various autoantibodies, which can determine several autoimmune diseases [38]. Some of these autoantibodies linked to the SARS-CoV-2 infection are anti-interferon antibodies (IFNs), lupus anticoagulant (LAC), anti-nuclear antibodies (ANA), rheumatoid factor (RF), p- and c-antineutrophil cytoplasmic antibodies (pANCA, cANCA), anticardiolipin antibodies (ACL), anti-Ro52 antibodies and anti-phosphatidylserine antibodies [38]. The precise role and mechanisms of autoantibodies in COVID-19, particularly in severe cases, remain unclear. However, research has postulated several ways in which SARS-CoV-2 can induce immune dysregulation:

1. The property of the virus to hyperstimulate the host’s immune system, leading to a prolonged hyperinflammatory state or autoimmune status;
2. Excessive formation of neutrophil extracellular traps (NETs), a pathological process defined as NETosis;
3. Molecular mimicry between the viral proteins and the host’s auto-(self-)antigens, determined by the biochemical similarity between these molecular elements [38].

According to Damoiseaux *et al*., in a report of the 15^th^ Dresden Symposium on Autoantibodies from 2021, antiphospholipid antibodies were represented mainly by IgG ACL (48%), followed by IgM ACL (21%), especially among COVID-19-positive patients. The same study revealed, however, that ACL were not associated with markers of thrombosis, an aspect described in antiphospholipid syndrome. However, IgG ACL were associated with worse disease severity and higher serum titers of ANA, regardless of the patient’s clinical status [38]. The same studies revealed that, among the COVID-19-positive patients, ACL were associated with high titers of anti-cytokine antibodies, thus leading to immune dysregulations.

In certain COVID-19 patients, molecular mimicry may play a significant role, as SARS-CoV-2 can elicit a broad array of antibodies that cross-react with viral proteins and the host's self-antigens (autoantigens) at the host level. Despite the limited research on the specific mechanisms of molecular mimicry in COVID-19, Vojdani *et al*. showed through ELISA that some monoclonal antibodies against the spike protein of SARS-CoV-2 react with various self-antigens, including glutamate-decarboxylase-65 (GAD-65), mitochondrial proteins, phospholipids, and hepatocyte microsomes [39]. However, other studies have not found evidence of the presence of autoantibodies associated with molecular mimicry that are commonly observed in classic autoimmune diseases, such as anti-mitochondrial antibodies (AMA), smooth muscle antibodies (SMA), anti-liver kidney microsomal antibodies (anti-LKM-1), anti-Scl-70, anti-RNA-polymerase III antibodies, SS-A and SS-B antibodies, and systemic lupus erythematosus-related autoantibodies, which have various pathogenic functions [38].

Chang *et al*. reported that hospitalized COVID-19 patients exhibit multiple IgG autoantibodies, some of which contribute to the formation of proinflammatory immune complexes, primarily on endothelial surfaces [40], potentially leading to inflammation in the blood vessels and thrombosis. For instance, an increased formation of NETs was found in COVID-19 patients with vasculitis. This was associated with a strong activation of neutrophils and the generation of proinflammatory NETs that contain nucleic acids, histones, and inflammatory peptides or proteins. Some of the autoantibodies discovered included anti-C1q antibodies, also seen in systemic lupus erythematosus, anti-β2GP1 antibodies, anti-bactericidal/permeability-increasing protein (BPI) antibodies, and anti-ACE-2 antibodies.

These molecular elements were recognized to exacerbate some pathophysiological phenomena observed in many severe COVID-19 patients, such as complement hyperactivation and the development of microthrombi. Furthermore, the studies mention that approximately 60-80% of all hospitalized patients with COVID-19 expressed at least one anti-centromere antibody (ACA) – also described in other autoimmune diseases, most frequently in CREST syndrome. Additionally, several other autoantigens were discovered, some of them being molecularly complexed with structural RNA, these molecular complexes serving as ligands for nucleic acid sensors, such as Toll-Like Receptors (TLR-7, TLR-3) in host cells. Furthermore, RNA and DNA molecules released from damaged tissues may form immune complexes with either viral proteins or autoantigens, stimulating autoantibody production and, eventually, exacerbating inflammatory phenomena [40].

Wang *et al*. conducted a study on the diverse functional autoantibodies in COVID-19 patients using the rapid extracellular antigen profiling (REAP) method. The REAP method revealed that multiple autoantibodies targeted various immune-related proteins and were elevated in severe COVID-19 patients, affecting lymphocyte function, leukocyte trafficking, interferon responses, type II immunity, and acute phase response. The study also found that IgG isotypes from patients with anti-GM-CSF, anti-CXCL-1, or anti-CXCL-7 autoantibodies could potentially block the signaling of these proteins. Furtermore, increased antibody-dependent cellular phagocytosis was observed in Raji B cells or Jurkat T cells due to the presence of anti-CD38 or anti-CD3ɛ autoantibodies [41]. These findings explained that autoantibodies reacting with immune-related proteins, as described in the study, could directly determine the inhibition of the activity of multiple cytokines and chemokines, but also to immune cell depletions in some severe COVID-19 patients.

Eric Y. Wang's research also revealed the pathogenic effects of tissue-associated autoantibodies in COVID-19 patients [41]. These autoantibodies targeted self-antigens found on various tissue components including vascular cells, plasma coagulation factors, and thrombocytes, as well as connective tissue components such as the extracellular matrix in internal organs like the lungs and tissues in the central nervous system, integumentary system, gastrointestinal tract, and other tissues. The study found that autoantigens like NXPH-1, PCSK-1, SLC2A10, and DCD correlated with markers of COVID-19 severity like D-dimers, ferritin, C-reactive protein, and lactate which can increase in severe COVID-19 [41].

Although autoimmunity appears to be essential in COVID-19, especially in the more severe forms of the disease, there is limited knowledge regarding the exact nature of PCS. Hence, the pathophysiology of PCS is yet to be unraveled. However, there are currently several studies that focus mainly on the immunopathological nature of the syndrome. This should not come as a surprise, given that the majority of signs, symptoms, and biological modifications revealed in COVID-19, especially in its severe forms, are determined by immune dysregulation or immunopathological phenomena, as described above. According to Ampudia *et al*., there is persistent autoimmune activation and a proinflammatory state in PCS; several autoantibodies were found in some patients with PCS, and it appears that the frequency of β2-glycoprotein-1 (β2-GP1) IgM autoantibodies, classically described in antiphospholipid syndrome, was higher in PCS patients compared with pre-pandemic controls [6]. The same study describes that, while 19 out of 33 patients, during the acute phase of COVID-19, expressed at least 1 autoantibody (latent autoimmunity), 11 out of 33 patients presented 2 or more autoantibodies (latent Poly autoimmunity), while in the context of PCS a number of 21 out of 33 patients expressed at least 1 autoantibody [6]. The presence of certain autoantibodies can impact the clinical and biological characteristics of PCS, potentially leading to overt autoimmunity [42]. Cañas suggests that the development of autoimmunity following COVID-19 recovery may be due to a temporary suppression of acquired and innate immunity that results in a loss of self-tolerance, or due to a form of immune reconstitution inflammatory syndrome (IRIS) in susceptible individuals [43]. This has been observed in some HIV-positive patients and results in an exaggerated immune response. According to Ampudia *et al*., PCS patients have higher levels of circulating naive B cells [6], which are a known source of autoantibodies. Furthermore, it should be noted that the same results showed that patients with PCS revealed very high levels of circulating proinflammatory cytokines, such as IFN-α, TNF- α, G-CSF, IL17A, IL-6, IL1-β, and IL-13, but also a decrease in interferon-γ-induced protein-10 (IP-10). Persistent IL-6 (one of the most important proinflammatory cytokines in COVID-19) dysregulation was found to be associated with generalized fatigue, sleeping difficulties, depression, and anxiety, as well as being one of the main proinflammatory molecules associated with the development of autoinflammatory reactions and autoimmunity, via pre-existing B-lymphocytes [30]. Other molecular elements found to contribute to the clinical presentation of PCS were IL-1β, TNF-α, IFN-γ, IL-10, IL-2, C-reactive protein, MCP-1, serum amyloid-A and metabolites of the kynurenine pathway [6] and, most probably, ferritin, which is currently recognized not only as a mere iron-binding protein but also as an important immunoinflammatory mediator [44].

One interesting pathophysiological aspect regarding the cellular immune responses in PCS resides in Ampudia’s report [6], which shows that 7 to 9 months after the remission of SARS-CoV-2 infection, most of the immune cellular components do not functionally return to their physiological state in PCS; it appears to be an atypical increase in CD4+ effector memory lymphocytes, CD8+ effector T lymphocytes, Th9-lymphocytes and in naive B lymphocytes. According to Diao B. *et al*., in acute COVID-19, there is a functional decrease in CD4+ and CD8+ T-lymphocytes and natural-killer cells [45]. This phenomenon is explained by the apoptosis determined by SARS-CoV-2 in lymphocytes that express ACE-2, with subsequent lymphopenia and CRS. In Ampudia’s study, it is revealed that lymphopenia persists in most patients with PCS [6]. Furthermore, a significant increase in Th9-lymphocytes was described [6]. Earlier studies showed that cellular responses mediated by Th9-lymphocytes, associated with IL-9 production, are pathophysiologically involved in immunoinflammatory phenomena, such as autoimmunity and asthma [46]. In 2020, Orologas-Stavrou *et al*. showed that a high Th9/Th17-lymphocytes ratio was associated with the persistence of a systemic inflammatory process (mediated by Th17-lymphocytes), particularly in the lung tissue (mediated by Th9-lymphocytes), 2 months after COVID-19 [47].

### Endocrine dysfunctions in PCS

Another presumed pathophysiological phenomenon that may explain the clinical and biological presentation of PCS is the dysfunction of the HPA axis. According to Pal R., in a study published in 2020 involving autopsies of several patients who died of COVID-19, there was evidence of viral genome, edema, and nerve cell degeneration found in the structure of the hypothalamus [48]. Furthermore, Pal R. and Wheatland proved that the proteins expressed by SARS-CoV (which shares similar structural patterns and the same ACE-2 receptors with SARS-CoV-2) are similar in their amino acid sequence to the biochemical structure of adrenocorticotropic hormone (ACTH), produced and released in the bloodstream by the pituitary gland [49,50]. According to studies, due to this particular biochemical similarity, the host’s immune system produces antibodies that interact not only with the SARS proteins but also with ACTH (molecular mimicry), which generates ACTH deficiency and secondary hypocortisolism (adrenal insufficiency).

The adrenal glands may also be directly affected, and the functional alteration of these organs may explain the non-specific clinical manifestations in some patients with PCS (abdominal pains, nausea, vomiting, fever, generalized fatigue, hypotension, confusion). However, the diagnosis of adrenal insufficiency is rarely detected or even suspected in post-COVID-19 patients. Several autopsy studies proved that epithelial cells of the adrenal glands undergo extensive necrosis after SARS-CoV (and SARS-CoV-2) infection. This process is possible due to the presence of ACE-2 receptors in the adrenal glands [51]. Furthermore, postmortem studies, as well as several case reports on patients with COVID-19, showed numerous microscopic adrenal lesions, adrenal hemorrhage, and adrenal infarction, with subsequent primary adrenal insufficiency [52-56], similar to Waterhouse-Friedrichsen syndrome in the context of fulminant meningococcemia.

### Post-COVID-19-associated malignancies

Although there is limited knowledge regarding the potential leukemogenesis activity of SARS-CoV-2, 3 case reports, described by Bruno Almeida Costa *et al*. in 2020 [57], describe the potential leukemogenesis developed secondary to SARS-CoV-2 infection, which suggests that post-COVID-19 syndrome may also manifest in the form of hematological malignancy, further complicating the clinical and biological presentation of this entity. These case reports describe the clinical and biological presentation of three adult patients, 35, 36, and 31 of age, respectively, each having had a recent SARS-CoV-2 infection, subsequently being diagnosed with acute lymphocytic leukemia (2 months after COVID-19 remission), myelodysplastic syndrome with an excess of blasts type 1 (approximately 2 months after COVID-19 remission) and acute myeloid leukemia (approximately 3 months after COVID-19 remission), respectively.

As studies show, SARS-CoV-2 binds to ACE-2 expressed by numerous cells in different tissues, including the bone marrow [58], which causes extensive downregulation of ACE-2. This phenomenon further promotes the enhanced activity of angiotensin-II through hyperactivation of the angiotensin-II receptor type 1 (AT1-R), which determines immune cell hyperactivation, as well as lung injury [59]. Given that ACE-2 has important physiological roles in regulating the renin-angiotensin-aldosterone system (RAS), the downregulation of ACE-2 following the binding of SARS-CoV-2 generates a systemic dysregulation of RAS. This disturbance also involves the cellular milieu of the bone marrow. An in vitro study from 2016 suggested that SARS-CoV-2-mediated dysregulation of the RAS system could promote the development of leukemia [60]. Angiotensin-II functions as a growth factor for acute myeloid leukemia cells and an anti-apoptotic factor for immune cells [61], thereby increasing the risk of hematological malignancies [62]. These findings were validated by experiments that showed the induction of apoptosis in myeloid leukemia cells treated with captopril, trandolapril, or losartan and in adult T-cell leukemia cells treated with telmisartan [63].

## CLINICAL AND BIOLOGICAL PRESENTATION

The clinical and biological presentation of PCS varies among patients and may generate differential diagnostic issues. The current general understanding of PCS shows that most patients express general and non-specific clinical manifestations, such as generalized fatigue, dyspnea, nausea, recurrent vomiting, hypotension, anosmia, and myalgias, but also cognitive impairments, defined under the name of brain fog, chest pain, insomnia, palpitations, diffuse arthralgias, neuropsychological manifestations, such as depression and/or anxiety. Other manifestations include fever, diarrhea, abdominal pains, skin rashes, loss of appetite, and changes in smell or taste [64].

From our personal clinical experience at the department of Internal Medicine [1], one adult male patient with no known associated comorbidities had a recent moderate-to-severe infection with SARS-CoV-2 and subsequently developed clinical and biological manifestations indicative of an inflammatory syndrome. Having had numerous admissions in our department for similar clinical manifestations, multiple differential diagnoses were performed over several months. The clinical and biological manifestations included generalized fatigue, diffuse arthralgias, fever associated with shivers, loss of appetite, nausea, very high serum levels of C reactive protein, marked hyperferritinemia, mild elevation of C3 complement fraction and slightly increased levels of rheumatoid factor, moderate-to-severe chronic anemia and a pulmonary nodule detected on chest X-ray and thoracic computed tomography (CT), associated with multiple mediastinal lymphadenopathies, with no diagnostic proof of an infectious process, autoimmune disease or malignancy, after multiple differential diagnoses. Lung cancer and pulmonary sarcoidosis were also excluded both clinically and biologically; paratracheal lymph node biopsy revealed the presence of lymph node necroinflammation and histiocyte infiltrates, with no associated malignant cells, which excluded lung cancer, and serum levels of angiotensin-converting enzyme (ACE) (an important serum marker in the diagnosis of sarcoidosis) were in the normal range, which suggested the absence of pulmonary sarcoidosis, which ultimately raised the suspicion of an obscure post-infectious inflammatory syndrome. The initiation of corticotherapy was the only treatment strategy that improved the patient's overall condition. High doses of methylprednisolone were effective in improving the patient's clinical and biological state, suggesting an underlying inflammatory condition. Given that the clinical state of the patient gradually began to deteriorate approximately one month after the remission of the viral infection and that the patient had no other known pathological condition, this resulted in the diagnosis of a post-COVID-19 inflammatory syndrome.

Biological modifications appear to be non-specific or indicative of an inflammatory syndrome. From our inpatient experience [1], persistent elevation of C reactive protein, chronic anemia, hyperferritinemia, and elevated D-dimers are frequently found in laboratory investigations. However, patients with signs and symptoms suggestive of adrenal insufficiency should be biologically examined for HPA dysregulation.

## DISCUSSION

PCS may be currently defined as a post-infectious systemic disorder, mainly inflammatory, due to several persisting pathophysiological phenomena which have their onset during the evolution of moderate and severe COVID-19. Immune dysregulation, including CRS and persisting hyperinflammation, as well as latent autoimmunity, appear to possess important pathogenic roles in the context of PCS, although other non-immunological mechanisms may be associated as well, including endocrine disturbances, such as HPA dysregulation and primary adrenal insufficiency.

The main pathophysiological mechanisms described in the present review may explain the origin of the systemic and somewhat non-specific clinical manifestations, which could generate multiple differential diagnostic issues. Patients who are known to have had a recent SARS-CoV-2 infection, who exhibit persisting or recurrent and non-specific clinical manifestations (fever, generalized fatigue, arthralgias, loss of appetite, myalgias, nausea, vomiting, confusion, insomnia, etc.), not associated with infections, malignancies, autoimmune or autoinflammatory disorders, should be suspected of PCS. Specific therapeutic management of PCS is yet to be described as pathophysiological mechanisms come to be understood.

From our clinical experience, the administration of glucocorticoids helped improve the patient’s clinical state and facilitated the decrease in the levels of C-reactive protein. However, it should be emphasized that long-term corticotherapy is known to be associated with adverse effects, including Cushing syndrome, osteoporosis, and immunosuppression, with an increased risk of infections. Thus, although corticotherapy may be an essential therapeutic strategy, pharmacological management in PCS requires further investigations and refinement to improve the patient’s quality of life.

## CONCLUSION

PCS is currently considered a clinically and biologically polymorphic entity, and its pathophysiology, although governed mainly by immunopathological or hyperinflammatory phenomena, requires refinement through future investigations to have clear diagnostic tools and a proper therapeutic or pharmacological strategy. Given that the clinical and biological presentation of PCS is polymorphic and may raise numerous differential diagnostic issues, internists should possess the main role in the management of PCS. Nevertheless, internists should collaborate with other medical specialists (rheumatologists, pulmonologists, hematologists, and intensive care physicians). The risk of mortality in PCS patients is currently unknown, as most cases appear to be either misdiagnosed or omitted. However, it may be assumed that the mortality risk is much higher in patients who exhibit hyperinflammatory-associated clinical manifestations and biological modifications (for example, elevated levels of C reactive protein, increased levels of D-dimers, hyperferritinemia), these being most probably associated with a high risk of evolution towards MODS.
